# Primary Care Patients’ Perspectives of Barriers and Enablers of Primary Prevention and Health Promotion—A Meta-Ethnographic Synthesis

**DOI:** 10.1371/journal.pone.0125004

**Published:** 2015-05-04

**Authors:** Patricia Moreno-Peral, Sonia Conejo-Cerón, Ana Fernández, Anna Berenguera, María Martínez-Andrés, Mariona Pons-Vigués, Emma Motrico, Beatriz Rodríguez-Martín, Juan A. Bellón, Maria Rubio-Valera

**Affiliations:** 1 Spanish Research Network for Preventive Activities and Health Promotion in Primary Care (RedIAPP), Barcelona, Spain; 2 Unidad de Investigación del Distrito Sanitario de Atención Primaria Málaga-Guadalhorce, Málaga, Spain; 3 Instituto de Investigación Biomédica de Málaga (IBIMA), Málaga, Spain; 4 Centre for Disability Research and Policy—Brain and Mind Research Institute, Faculty of Health Sciences, The University of Sydney, Sydney, Australia; 5 Institut Universitari d’Investigació en Atenció Primària Jordi Gol (IDIAP Jordi Gol), Barcelona, Spain; 6 Universitat Autònoma de Barcelona, Bellaterra, Barcelona, Spain; 7 Social and Health Care Research Center, University of Castilla-La Mancha, Cuenca, Spain; 8 Departamento de Psicología, Sociología y Trabajo Social, Universidad Loyola Andalucía, Sevilla, Spain; 9 Centro de Salud El Palo, Málaga, Spain; 10 Departamento de Medicina Preventiva y Salud Pública, Universidad de Málaga, Málaga, Spain; 11 Research and Development Unit, Fundació Sant Joan de Déu, Esplugues de Llobregat, Barcelona, Spain; 12 School of Pharmacy, Universitat de Barcelona, Barcelona, Spain; University of Louisville School of Medicine, UNITED STATES

## Abstract

**Background:**

Primary care (PC) patients have difficulties in committing to and incorporating primary prevention and health promotion (PP&HP) activities into their long-term care. We aimed to re-interpret, for the first time, qualitative findings regarding factors affecting PC patients' acceptance of PP&HP activities.

**Methods and Findings:**

A meta-ethnographic synthesis was generated following electronic and manual searches that retrieved 29 articles. Papers were reviewed and translated to produce a re-interpretation of the extracted concepts. The factors affecting PC patients' receptiveness to PP&HP activities were framed in a four-level ecological model (intrapersonal, interpersonal, institutional and environment and society). Intrapersonal factors (patients' beliefs/attitudes, knowledge, skills, self-concept, motivation and resources) were the most numerous, with almost 25 different factors. Public health education to modify erroneous beliefs and values regarding PP&HP could encourage a transition to healthier lifestyles. Health care professionals' abilities to communicate and involve patients in the decision-making process can act as facilitators. Biopsychosocial training (with emphasis on communication skills) for health professionals must start with undergraduates. Increased consultation time, the use of reminders, follow-up visits and tools for communicating risk and motivating patients could be applied at the intrapersonal level. Collaborative care involving other health professionals (nutritionists or psychotherapists) and family and community stakeholders (teachers or gym trainers) was important in developing healthier habits. Patients also cited barriers related to the built environment and socioeconomic difficulties that highlighted the need for policies promoting social justice and equity. Encouraging PP&HP using social marketing strategies and regulating media to control its impact on health were also cited. Only the perspectives of PC patients in the context of chronic conditions were considered thus limiting extrapolation to other contexts.

**Conclusions:**

Several factors affect PP&HP. This must be taken into account when designing PP&HP activities if they are to be successfully implemented and maintained in routine practice.

## Introduction

Primary care (PC) is the area of the health care system most suited to offering primary prevention and health promotion (PP&HP) activities as it is easily accessible, provides continuity of care and is used by a large proportion of the population [1,2]. PP& HP activities include initiatives to maintain or increase the level of wellness and to reduce risk factors associated with distinct diseases through the promotion of lifestyle changes (e.g. healthy eating, physical activity or smoking cessation) and prevention of physical and mental disease such as cardiovascular diseases or depression. Existing evidence supports the effectiveness and benefits of PP&HP in PC [3,4]. However, it is well known that PC professionals have difficulties in implementing these activities and patients can sometimes struggle to engage in them [5–8].

In a previous meta-ethnography, published qualitative research on the primary care physicians and nurses’ perspectives of the facilitators and barriers to applying PP&HP interventions to PC patients was synthesized. [9]. A five-level ecological model was designed to fit the following barriers and facilitators affecting PC professionals: intrapersonal (professionals’ beliefs about PP&HP, experience, skills, knowledge, and self-concept); interpersonal (patients' attitudes and behavior with respect to PP&HP and PC professionals relationship with specialists, health center managers and staff); institutional (workload, time limitations, referral options, biomedical-model predominance); community (social and cultural backgrounds of the population served, local referral resources, mass-media messages, pharmaceutical-industry campaigns, and the relative importance of PP&HP in university curricula); and public policy (private and public health-system models).

Knowing which factors affect PC professionals' implementation of PP&HP is important if they are to be successful. Patients have to face distinct barriers when implementing lifestyle changes. Patients' preferences and values are among the basic principles of patient-centered care [10]. In the context of this care model, increasing our understanding of patients' perceptions regarding PP&HP will help to engage patients in the care process, promote their autonomy, empower them and improve continuity of care [11,12]. A number of qualitative studies have been carried out with the aim of determining the perceptions of PC patients with respect to primary prevention of specific chronic diseases and the promotion of different health activities or lifestyle modification factors [5,8,13,14]. These studies have highlighted multicomponent factors among the barriers to implementation of PP&HP, such as cultural issues, lack of time with the health professional, and patients' demotivation or doubts about professionals’ ability to carry out these activities.

These barriers hamper the implementation of PP&HP activities and limit effectiveness in the actual clinical practice of PP&HP. Consequently, it is essential to generate an explanatory framework of the main barriers and facilitators with the aim of adapting the interventions to minimize barriers and maximize facilitators. The main goals are to improve the implementation, acceptability, effectiveness and maintenance of PP&HP activities in real primary care clinical practice.

PC patients and professionals play an important role in PP&HP activities. To the best our knowledge, there is no synthesis in the literature on the major barriers and facilitators in PP&HP in PC as perceived by patients. Hence, we aim to identify and synthesize, using the meta-ethnographic technique, available qualitative research into barriers and facilitators identified by PC patients to develop PP&HP activities.

## Methods

A meta-ethnographic approach, as developed by Noblit and Hare, 1988 [15] and adapted by Britten and colleagues, 2002 [16], was used to synthesize the available evidence. Information was extracted, re-interpreted and aggregated to develop a novel contribution to the literature.

The procedures used to complete this synthesis followed the methods used in a previous review conducted by the research team to synthesize qualitative research into barriers and facilitators identified by primary care physicians and nurses in the implementation of PP&HP [9].

### Research Question

The central research question was: According to published qualitative research, what are the barriers and facilitators (phenomena of interest) to engaging adult primary-care patients in primary prevention and health-promotion activities?

### Study Search

Two researchers (PMP and SCC) separately searched, from inception to February 2014, the PubMed, Web of Science, Global Health and CINHAL electronic databases. Terms included in the search related to qualitative research were risk reduction and PP&HP, and patients. The search strategies prioritized sensitivity over specificity which increases the chances of identifying both relevant citations and non-relevant citations (S1 Table).

To retrieve papers that could have been missed in the electronic search, we reviewed the references of articles included in the synthesis and invited primary care professionals and researchers from the Spanish “Research Network for Preventative Activities and Health Promotion in Primary Care” (RedIAPP) [17] to suggest relevant papers.

### Inclusion Criteria and Study Selection

Papers identified in the search were screened independently by two researchers (PMP and SCC) by reviewing the title and abstract. Full-text selection was made in duplicate by PMP or SCC and AB, AF, BRM, EM, MMA, MPV or MRV. In case of disagreement, a third researcher was consulted.

We included papers written in English, Spanish or Portuguese that explored the perceptions of primary care patients using qualitative methods for data collection and analysis. Papers using qualitative methods for data collection but used quantitative analytical strategies were excluded. When mixed methods were used in the research, we included them if the qualitative results were described and discussed independently of the quantitative findings. The focus of the research had to be primary prevention of chronic conditions or health promotion (lifestyle changes) in adults. We excluded studies dealing with primary prevention of health promotion in children or adolescents, secondary or tertiary prevention, prevention of acute diseases (e.g. infections) or vaccines. Papers interviewing patients from settings other than primary care were only included if primary prevention and health promotion activities were discussed in the context of primary care. When patients were interviewed together with physicians or other professionals, the papers were excluded when it was not possible to clearly identify patients’ opinions.

### Quality Appraisal

The appropriateness of using checklists to evaluate the quality of qualitative research studies continues to be an issue of concern and it is recommended that categorizing qualitative papers according to a score derived from a checklist be avoided [18–20]. As in previous qualitative syntheses [9,21] quality was discussed in terms of research coherence, relevance and utility of findings, taking into consideration the suitability of the design with respect to the research question, data collection procedures, rigor of analysis and presentation of primary data [7,22]. The papers were reviewed independently by two team members and the quality of each paper was discussed taking these quality criteria into account. No paper was excluded due to quality issues.

### Data Abstraction

**Fig 1:**
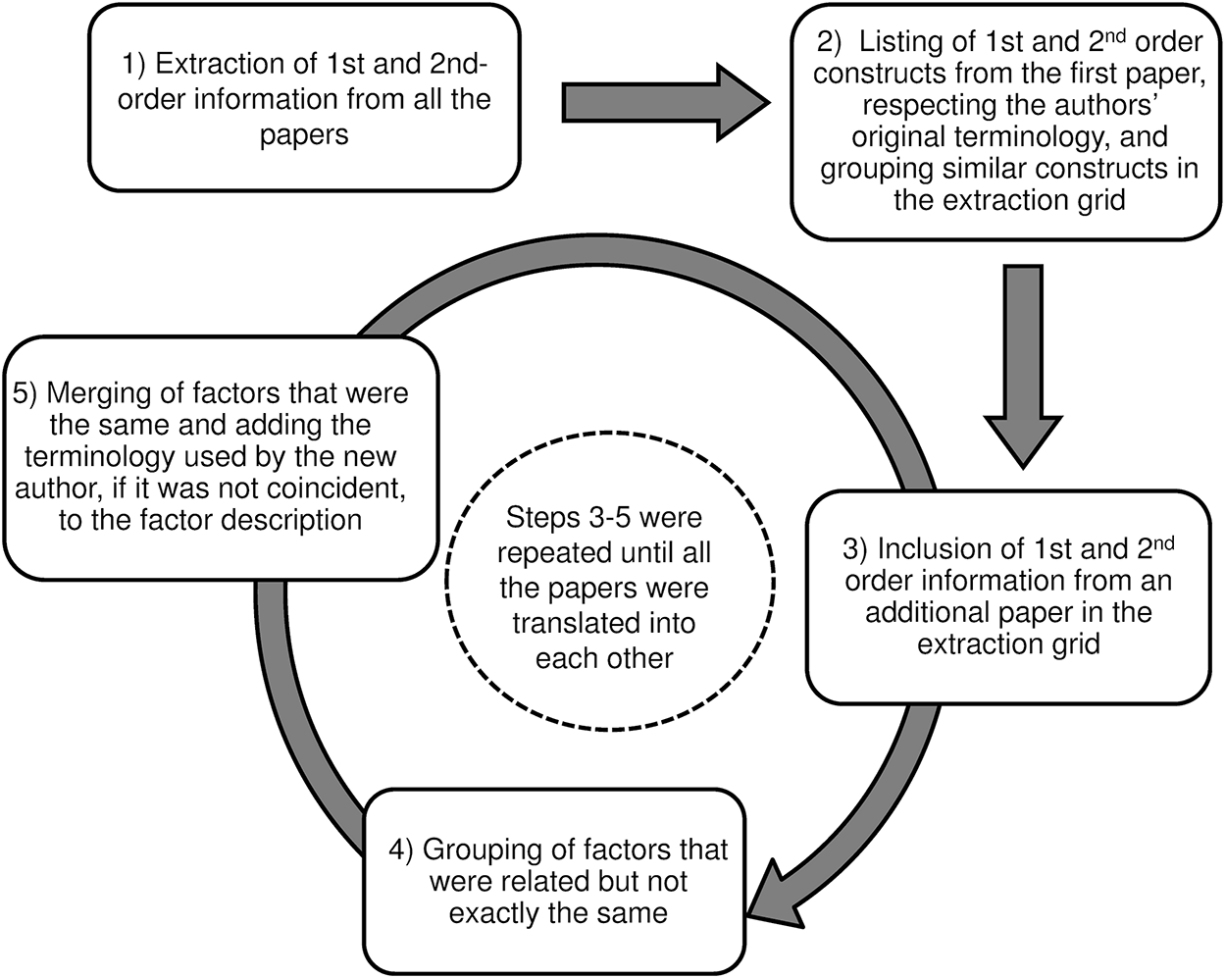
Translation and synthesis of data from original papers.

Fig 1 schematizes the data synthesis process. A data abstraction form was used to extract study characteristics (methodology and sampling characteristics) and key findings in duplicate by PMP or SCC and AB, AF, BRM, EM, MMA, MPV or MRV. One of the papers could only be extracted by one researcher because it was written in Portuguese. The abstraction form allowed separate extraction of first-order constructs (quotations describing factors identified by the primary care patients originally interviewed in the paper), second-order constructs (interpretations based on the patients quotations made by the authors of the original studies) and comments or interpretations from our research team based on the first and second-order constructs that could be used to re-interpret the aggregated data.

### Translation of Papers

After all studies had gone through the process of data abstraction, papers were read again by PMP and SCC using the abstraction forms to complete a table where first and second-order constructs were listed and grouped. To guide the process, we used an ecological model similar to the one used to elaborate the model of the factors affecting the implementation of PP&HP activities by primary care professionals described above [9].

First and second-order information was grouped and mapped following this model. Papers were reviewed one at a time and translated, unifying the interpretations of the different authors when they referred to the same factor, through a process of constant comparison and extraction of information piece by piece (see Fig 1).

To begin the process, we listed the first and second-order information from the first paper, respecting the authors' original terminology. Subsequently, we extracted the findings from the next study grouping similar constructs and adding the terminology used by the second author for the same factor to its description. We extracted factors that were related but not identical separately but put them together in the extraction grid.

The process described above was repeated for each of the retrieved papers until they were all translated [16].

The new interpretations and relationships between concepts (third-order constructs) that emerged during the translation process were recorded in the grid to inform the re-interpretation of the data.

Finally, all the authors reviewed the results of the translation and aggregation of the studies to check that all the first and second-order information that they had extracted from the original papers during the data abstraction process were included in the grid and had been adequately translated.

### Third-order synthesis

For the third-order synthesis we re-interpreted the synthesis of the first and second-order constructs [16]. The factors and groups of factors were reviewed and refined, identifying relationships between categories of factors, re-organizing the information and modifying the grid structure. We used a line-of-argument synthesis to produce a reconceptualization of the findings that fit an ecological model where factors affecting the implementation of PP&HP moved from a micro to a macro level: intrapersonal, interpersonal, institutional and environment and society factors [23]. This was done by PMP, SCC and MRV and discussed by all the authors.

## Results

### Studies Identified

**Fig 2:**
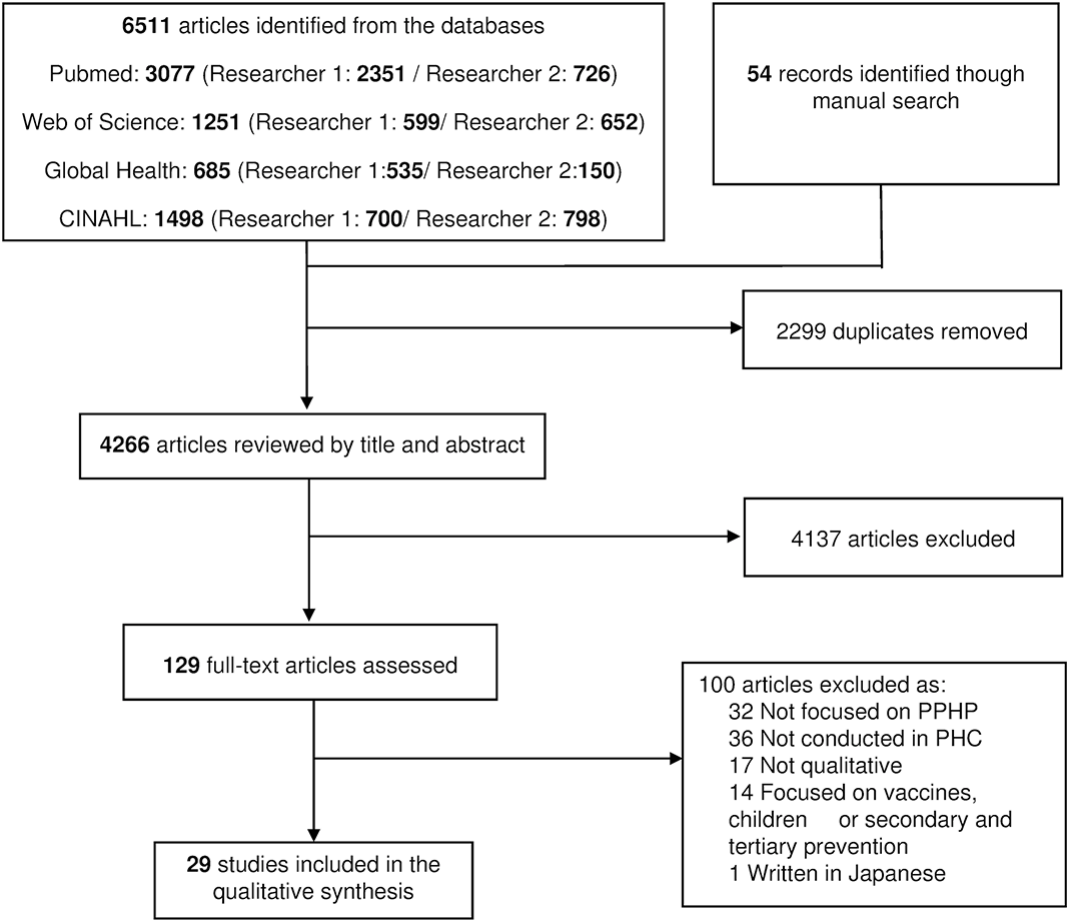
Flow-chart of the systematic review.

Fig 2 shows the search and study-selection flow chart. The search strategy and manual search retrieved 6,565 potentially relevant articles. Of these, 2,299 (35%) were duplicates (indexed in more than one database). Finally, a total of 29 (0.4%) publications were selected (Fig 2) [5,6,8,13,14,24–47]. The data from these studies were extracted and included in Table 1.

**Table 1:** Study characteristics.

	Study	Fieldwork year	Country	Participants	Age range	Number of females (%)	Data collection technique	Aim (using original study wording)
1	Bellón 2014 [24]	2009	Spain	52	18–75	27 (52%)	Focus groups	To explore patients’ opinions towards receiving information about their risk for depression and the values and criteria upon which their opinions are based.
2	Costello 2013 [25]	_	USA	31	≥60	11 (35%)	Focus groups	To explore the perceptions of independent living older adults regarding their physicians' role in promoting physical activity.
3	Elwell 2013 [5]	_	UK	7	40–74	5 (71%)	Focus groups	To examine patients and health professionals perspectives on lifestyle behaviour change and to inform the development of a lifestyle behaviour change intervention to be used in primary care.
4	Lu 2013 [26]	2012	Australia	18	40–69	11 (61%)	Semi-structured telephone interviews	To explore patients’ views on risk, assessment and their general practitioner’s role, and how these factors may impact their uptake of preventive care.
5	Calderon 2011 [27]	2006	Spain	15	45–80	7 (47%)	Discussion groups	To gain an in-depth understanding of general practitioners’ and patients’ perceptions abouthealth promotion and prevention in primary health care, and to define the areas that could be improved in future interventions.
6	Dhanapalaratnam 2011 [28]	_	Australia	20	50–69	12 (60%)	Semi-structured telephone interviews	To explore the factors contributing to sustain or no sustain behaviour change following a lifestyle intervention in general practice.
7	Gale 2011 [13]	2003	UK	17	40–79	2 (12%)	Interviews	To identify and explore the attitudes of patients and general practitioners towards preventative medication for cardiovascular disease after they have received information about it; to identify implications for practice and prescribing.
8	Ingram 2011 [29]	2007	USA	33	44–69	33 (100%)	Focus groups	To elicit recollections of the women’s outcome expectations and the barriers they experienced; to obtain feedback on all of the physical activity program components (walking prescription, workshops, tailored telephone contacts), and seek suggestions for changes to make the program more appealing to Afro-American women.
9	Mazza 2011 [8]	2010	Australia	85	>25	41 (48%)	Focus groups	To identify barriers to, and enablers of, the uptake of preventive care in general practice from the perspective of community members, and to explore their sense of the effectiveness of that care.
10	Walseth 2011 [30]	_	Norway	12	teenage-60	5 (42%)	Qualitative observation and interview	To elucidate the relevance of Habermas’s theory as a practical deliberation procedure in lifestyle counselling in general practice, using a patient perspective; and to search for topics which patients consider of significance in such consultations.
11	Costa 2010 [31]	2009	Brazil	_	_	_	Semi-structured interviews	To identify the users' perception of group-experienced health promotion practices in a family health basic unit.
12	Horne 2010 [14]	2009	UK	127	60–70	81 (64%)	Focus groups and depth semi-structured interviews	To explore the influence of primary health care professionals in increasing exercise and physical activity among 60–70-year-old White and South Asian community dwellers.
13	O’Sulivan 2010 [32]	_	Canada	15	32–65	11 (73%)	Individual, semi-structured interviews (each participant took part in three interviews)	To understand why the Physical Activity Counseling intervention worked and the patient perspective of the counseling. Also, to explore the experiences, thoughts, and feelings of the patients who received both the brief and intensive arms of the counseling intervention.
14	Wolf 2010 [33]	_	USA	234	30–70	117 (50%)	Focus groups	(i) What are the barriers that people with different information-seeking orientations have in receiving healthy lifestyle and disease prevention messages in the primary care setting? (ii) where are the windows of opportunity for prevention counseling during the office visit and do these differ by information-seeking styles? And (iii) what are the desired aspects of prevention counseling that people hope to receive from their healthcare provider?
15	Figueira 2009 [34]	2007	Brazil	20	18–37	20 (100%)	Semi-structured interviews	To analyze perceptions and participation of female users of basic health units with regard to disease prevention and health promotion
16	Kehler_a 2008 [35]	_	Denmark	12	42–74	2 (17%)	Individual interviews	To explore and describe motivational aspects related to potential lifestyle changes among patients at increased risk of cardiovascular disease following preventive consultations in general practice.
17	Kehler_b 2008 [36]	_	Denmark	12	42–74	2 (17%)	Individual interviews	To explore and analyze experiences of preventive consultations in patients at high cardiovascular risk.
18	Elley 2007 [37]	2003	New Zealand	15	43–78	9 (60%)	Semi-structured telephone interviews	To explore attitudes and subjective experiences of those who received an intervention of physical activity promotion.
19	Goldman 2006 [38]	2003	USA	50	27–84	21 (42%)	Focus groups	To explore patients’ perceptions of cholesterol and cardiovascular disease risk and their reactions to 3 strategies for communicating cardiovascular disease risk.
20	Lundqvist 2006 [39]	_	Sweden	9	47–70	9 (100%)	Interviews	To examine attitudes and barriers to smoking cessation among middle aged and elderly women.
21	Ribera 2006 [40]	2001	Spain	20	28–48	17 (85%)	Focus groups, semi-standardized individual interviews and short individual interviews	To explore experience-based information and generate explanations for the lack of micro-level integration of promoting physical activity in general practices of Barcelona.
22	Bowden 2004 [41]	2001–2002	USA	74	21–83	63 (85%)	Focus groups	To report the results of an intervention program to help rural adults change their health risk behaviours and to describe the barriers to behavioural change in the rural environment, as expressed by rural adults in focus group discussions.
23	Stermer 2004 [42]	2002	UK	18	_	_	Focus groups	To explore the views and opinions of patients with a family history of colorectal cancer, and of primary and secondary care health professionals, on how to improve current services for individuals with a family history of colorectal cancer.
24	Van Steenkiste 2004 [43]	_	Netherlands	22	40–70	5 (23%)	Semi-structured interviews	To explore those barriers that impede effective communication on cardiovascular risk and prevention during consultations in primary care.
25	Fuller 2003 [6]	_	UK (Scotland)	30	_	15 (50%)	Semi-structured interviews	To investigate the views of general practitioners and their patients about healthy eating and the provision of healthy eating advice in general practice.
26	Butler 1998 [44]	_	UK (Wales)	42	≥20	24 (57%)	Semi-structured interviews	To determine the effectiveness and acceptability of general practitioners' opportunistic antismoking interventions by examining detailed accounts of smokers' experiences of these.
27	Dilloway 1998 [45]	_	UK	19	20–60	19 (100%)	Semi-structured interviews	To examine female patients' concerns and experiences in relation to a number of sexual health promotion issues.
28	Cogswell 1993 [46]	1984–1986	USA	322	≥18	193 (60%)	Focus groups	To explore health care consumers’ perspectives on provision of preventive care by physicians.
29	Stott 1990 [47]	1987	UK (Wales)	130	25–40	130 (100%)	Semi-structured interviews	To explore the women´s view in the role that the primary care team could and should play in health promotion. To compare quantitative and qualitative data.

Of the 29 studies, 21 (72.4%) had been published from 2006 onwards. Most of the studies had been conducted in the UK (8; 28%), USA (6; 21%), Australia (3; 10%) and Spain (3; 10%). The number of participants in the studies included ranged from 7 to 322. The main data-collection techniques used were individual semi-structured interviews and focus groups.

### Quality Appraisal

The analysis strategy was poorly described in many studies although it seemed appropriate. The author's own position was rarely presented or discussed. In several studies the results were not checked with participants (respondent validation) which could have affected the validity of the results.

In all papers, qualitative research was appropriate for the question posed and the methods of data collection were also appropriate. Overall, the sampling strategy was adequate and the studies followed methodologically rigorous procedures. Presentation of data was clear and systematic and the results were credible and illustrated by quotations. The conclusions were consistent with the results and were supported by the evidence presented.

### Synthesis

**Fig 3:**
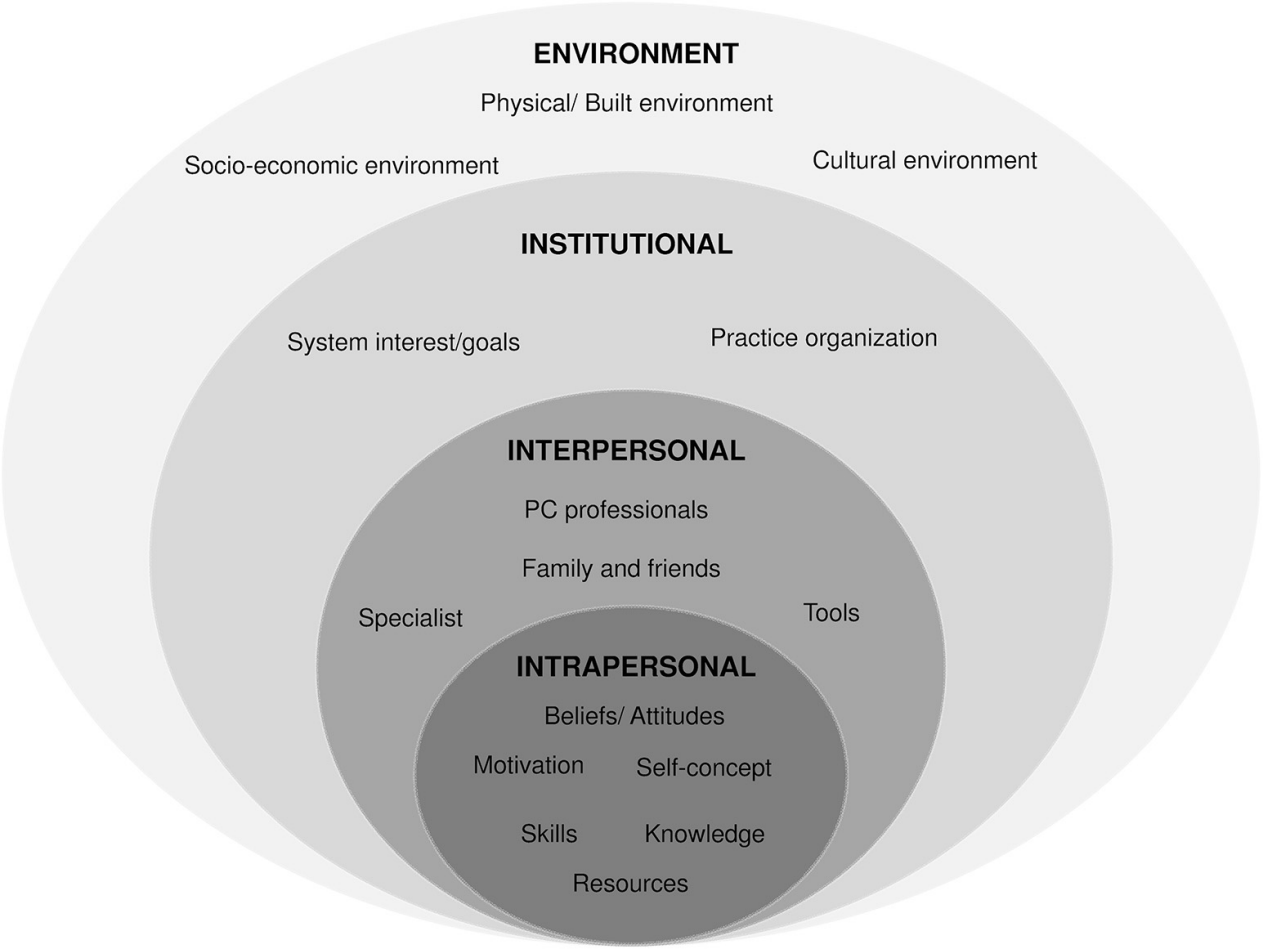
Ecological model of the factors affecting the implementation of PP&HP activities by primary care patients.

The factors identified by PC patients as relevant to the development of PP&HP activities can be described in a four-level ecological model [23] (Fig 3). Higher (or macro) levels could affect lower (or micro) levels while factors at the same level could affect each other.

The translation of the first and second-order constructs to the third-order synthesis (construction of the ecological model) can be seen in Table 2.

**Table 2:** Translation of 1st and 2nd order constructs and interpretation through 3rd order constructs and sources.

3rd order FACTORS	3rd order constructs	2nd order constructs (translated)	Sources
INTRAPERSONAL factors	Beliefs/Attitudes	Risk is out of (within) patient control (External/Internal locus of control). Prevention is (not) patients' responsibility	5,24,26,32,33,36,38,43,44,47
		PP&HP is only necessary in high risk patients (genetic inheritance or family history or those with concomitant risk factors) or when there is a perception of symptoms that affect patients' health	6, 8, 26, 40
		PP&HP is a passing trend	38
		Some unhealthy lifestyles favor mental health ("Everyone deserves to indulge occasionally")	6
		It does not makes sense in the elderly (it is too late)	5
		Fear of side effects of PP&HP (side effects of statins, potential injuries when doing physical activity, etc.)	13, 14
		There are exceptions to the rule (i.e. giving examples of people following unhealthy habits with no negative consequences for their health)	27, 43
		Comorbid physical and mental illnesses hamper the adoption of lifestyle changes	26, 28, 29, 35, 37
		Knowing risk is only necessary/interesting if there is a treatment to prevent the disease	24
		"Ostrich strategy" (The patient prefers not to know about risk)	24, 33, 38, 43
		Lack of trust in risk factors as predictors of disease. Lack of trust in the effectiveness of PP&HP activities in preventing disease.	13, 24, 27, 35, 38, 43
		Empirical evidence of risk (e.g., test showing deviated blood markers)	26, 43
	Knowledge	Positive (and negative) consequences of (un)healthy habits (e.g., smoking, physical activity, etc.)	26, 27, 37–41, 43, 44, 46
		(Lack of) knowledge about what lifestyle changes to make and how, and where to find guidance and advice	14, 35, 36, 38, 40, 41
	Skills	Patients' ability to find information on PP&HP activities	47
		Capacity to understand risk indicators	38, 43
		(In)ability to remember professionals' advice	43
	Self-concept	Self-esteem, self-efficacy and self-confidence	5, 28, 31, 32, 34, 37, 39, 41
	Motivation	(Lack of) motivation and interest	5, 13, 14, 26, 27, 31, 32, 36, 39, 40, 43
		Aspects that improve motivation (threat of potential disease, patients' guilt, perception of quick improvements when making a lifestyle change)	26, 27, 37, 44,
		Positive reinforcement of unhealthy habits	34, 36
		Difficulties in maintaining lifestyle changes over time	26
	Resources	Patients' lack of time (workload and/or family commitments)	5,8, 26, 28, 29, 32–35, 37, 41
		Lack of financial resources (cost of PP&HP activities)	8, 26, 32–34, 41
INTERPERSONAL	PC professionals (and other PC staff)	(Lack of) trust in provider (training, motivation, attitude, knowledge about available resources, communication skills) and care provision characteristics (instilling fear, inadequate treatment or support, contradictory messages)	5, 6, 8, 13, 14, 24–28, 30,32, 33, 36–42, 44–47
		Importance given by PC professionals to PP&HP in the elderly	14
		Judgmental professionals as an invasion of patients' independence	45
		Advice as an invasion of patients' privacy (the professional nags the patient; trotting out usual advice and preaching)	6, 27, 44
		Limited influence (PC professionals' interventions are simple tips and/or ineffective, what they can do is insufficient)	27, 32, 44
		Good patient-PC professional relationship	8, 27, 30, 32, 36, 47
		Patient-centered care (advice adapted to patient’s circumstances, personalized care) and shared decision-making	5, 13, 24, 26, 27, 32
		The patient makes a commitment with the professional to reach agreed health goals	30
		Biomedical model (Risk is not a disease, the GPs should focus on diagnosis and treatment)	6
		PP&HP is the responsibility of PC professionals (must provide information on which PP&HP activities to perform and how do them)	43
		PC professional has a lot of burden, they are too busy	6, 8, 13, 31, 42, 45, 47
		Tools to facilitate the communication of risk and patient education and as an excuse to initiate patient evaluation of risk	38, 45
		Use of communication technology (email, sms, etc) to send reminders and support messages	14
		(Lack of) Reminders, follow-up visits and assessment of results	5, 14, 28, 32
	Other professionals and specialists	Support from specialists and professionals in specific activities (e.g., nutritionist or physical trainer)	14, 29, 32, 36, 37, 39
	Family and friends	Social support and support from peers	8, 14, 24, 28, 34, 41
		Pets entail commitment to perform physical activity	37
INSTITUTIONAL	Organization of physicians' practice	Waiting lists	33
		Professionals’ lack of time	5, 8, 13, 30, 31, 36, 47
		(Lack of) resources for treatment, follow-up and referral (support groups, nutritionists, prescribing exercise)	14, 27, 29, 31–33, 36, 39
	System interests/goals	Private and public health institutions do not promote or cover PP&HP because it is unprofitable	14, 31, 33, 39–41, 46
		PC health centers are (not) an adequate reference point for PP&HP	34, 42
ENVIRONMENT AND SOCIETY	Physical context	Built environment (e.g., bike lanes, parks or pedestrian paths)	29, 39, 41
	Cultural context	Dietary traditions and other cultural lifestyle habits	27, 29, 39, 41
		Social norms that incentivize (un)healthy habits	27, 28
		Social stigma of unhealthy habits (e.g., alcohol consumption, promotion of sexual health, etc.)	27, 45
		Mass media impact	6, 38, 42, 46
		Lack of focus on PP&HP in health professionals’ university training	31, 40
	Socio-economic context	Lack of public policies that promote PP&HP	6, 8, 40, 42, 46
		Lack of work/personal-life balance	33

#### Intrapersonal factors

The factors at this level are: patients' beliefs/attitudes [5,6,8,13,14,24,26–29,32,33,35–38,40,43,44,47], knowledge [14,26,27,35–41,43,44,46], skills [38,43,47], self-concept [5,28,31,32,34,37,39,41], motivation [5,13,14,26,27,31,32,34,36,37,39,40,43,44] and resources [5,8,26,28,29,32–35,37,41].

Several beliefs and attitudes could act as barriers to the implementation of PP&HP activities: prevention is not patients' responsibility or escapes their control [5,24,26,32,33,36,38,43,44,47]; PP&HP activities are only necessary when there is a high risk (e.g., inherited risk) or perception of symptoms affecting patients' health [6,8,26,40]; or PP&HP is a passing trend [38]. Other beliefs can serve as an excuse to perpetuate unhealthy lifestyles: people need to indulge occasionally to maintain good mental health [6]; PP&HP is senseless for the elderly [5]; or PP&HP have adverse effects (side effects of statins, injuries when exercising, etc) [13,14]. The fear of side effects can give rise to situations where high credibility is given to exceptions to the rule (e.g., giving examples of people following unhealthy habits who never fell ill) [27,43].

According to patients, physical and mental comorbid illnesses hamper the adoption of lifestyle changes. Comorbid illnesses are accompanied by pain and/or emotional suffering that demotivates the person and complicates the implementation of PP&HP activities [26,28,29,35,37].

With regard to risk, some patients believe that it only makes sense if something can be done to prevent the health problem [24] while others prefer not to know their degree of risk even when something can be done to minimize it ("ostrich strategy") [24,33,38,43]. This could be related to the lack of trust in risk factors as predictors of disease or lack of faith in the effectiveness of PP&HP activities [13,24,27,35,38,43]. Patients believe that empirical evidence of risk (e.g., blood tests) can increase their trust in PP&HP activities and motivate them [26,43].

Patients' knowledge and skills can also influence and modify their beliefs and attitudes. Increasing knowledge of the negative consequences of unhealthy habits and the benefits of healthy ones can motivate patients to accept PP&HP [26,27,37–41,43,44,46]. Conversely, lack of knowledge about what lifestyle changes to adopt (e.g., diet) and how to (i.e. what food is healthy, where it can be found and how it should be cooked) as well as being unaware of sources of guidance and advice can limit patients capacity to implement PP&HP and negatively affect their motivation and attitude [14,35,36,38,40,41].

Patients' ability to find information about PP&HP activities without the help of PC professionals [47], their capacity to understand risk indicators (that tend to be complex) [38,43] and to remember health advice can play a role in the implementation of these activities [43]. Similarly, self-concept [5,28,31,32,34,37,39,41] and motivation to change [5,13,14,26,27,31,32,36,39,40,43] can also influence lifestyle modifications. Some of the retrieved studies revealed a wide-ranging construct, self-concept, which includes self-esteem, self-efficacy and self-confidence. Many aspects can improve motivation: the threat of disease, patients' feelings of guilt and sense of responsibility, or the perception of improvement when performing PP&HP activities [26,27,37,44]. However, some unhealthy habits produce positive reinforcement [34,36] and patients may experience difficulties in maintain motivation and lifestyle changes over time [26].

Patients' resources also condition the implementation of lifestyle changes. Workload and family commitments reduce patients' time and energy [5,8,26,28,29,32–35,37,41]. Furthermore, some PP&HP activities can require expenditure (e.g., sports centre). Lack of financial resources is consequently perceived as another barrier to PP&HP activities by patients [8,26,32–34,41].

#### Interpersonal factors

According to patients, other people affect their implementation of PP&HP activities: PC professionals [5,6,8,13,14,24–28,30–33,36–47], specialists [14,29,32,36,37,39], and family and friends [8,14,24,28,34,37,41].

PC professionals play an important role in promoting or impeding patients’ implementation of PP&HP. This is influenced by their training, motivation, attitudes towards PP&HP, communication skills and knowledge about available resources for the patient. Patients perceive that some PC professionals give no importance to PP&HP in the elderly and do not promote lifestyle changes in this population group [14]. If PC professionals instill fear, provide treatment or support that patients consider inadequate or transmit contradictory messages to the patient, the patients’ trust in the providers can diminish which may affect professionals’ capacity to promote lifestyles changes [5,6,8,13,14,24–28,30,32,33,36–42,44–47]. If patients feel that they are being judged by the healthcare professionals or that they are putting pressure on them and simply trotting out the usual advice [6,27,44], they can feel that their independence is threatened [45]. The resulting rebound effect can limit adherence to advice and damage the patient-provider relationship. Furthermore, some patients consider that the degree of influence of PC professionals is limited and that their interventions are just tips. These are insufficient to have an impact on prevention of diseases or health promotion [27,32,44]. A good relationship between the patient and the professional [8,27,30,32,36,47], the use of a patient-centered care approach (with messages personalized according to patients’ circumstances and shared decision-making) [5,13,24,26,27,32] and patients' commitment to the professional and the agreed goals can facilitate the implementation of PP&HP activities [30].

The relationship with PC professionals is also affected by the health model. Some patients believe that the aim of PC professionals should be detection and treatment of disease [6]. These patients give little or no importance to PP&HP. On the other hand, some patients think that PP&HP are among the responsibilities of PC professionals and they should provide information about PP&HP activities as well as detailed information on how to develop them [43]. However, patients perceive that PC professionals already have a heavy workload and that this interferes with the provision of PP&HP [6,8,13,31,42,45,47].

Use of tools can facilitate the communication of risk, improve patient education and help patients to benefit from advice. These tools include visual materials, information and communication technology, reminders and follow-up assessment visits. The use of visual tools for communication of risk can facilitate patients’ comprehension and serve as an excuse to initiate risk evaluation in delicate or complex topics [38,45]. Email and short message services (sms) can be used to send reminders or support messages to motivate the patient [14]. Other reminders, follow-up visits and assessment of improvements in patients' health can help to maintain patients’ motivation [5,14,28,32].

Whether they work at the PC centre or not, other professionals can facilitate patients’ implementation of PP&HP. Specialists and experts on the specific activities (e.g., nutritionists or physical trainers) can improve patients’ knowledge of PP&HP activities as well as their skills, attitudes and motivation [14,29,32,36,37,39].

Social support from family and friends can also increase patients’ motivation to take up these activities. Their approval and encouragement can facilitate lifestyle changes in the patient (e.g., smoking cessation as a commitment to grandchildren) and changes can be more easily maintained over time if the activity is shared with a peer (e.g., an exercise group) [8,14,24,28,34,41]. Finally, pets can also promote healthier lifestyles by through commitment to some of these lifestyle changes (e.g., physical activity) [37].

#### Institutional factors

Aspects of the PC and wider health system organization are perceived by patients as affecting both them and PC professionals. Long waiting lists [33] and demands on professional’s time are perceived as barriers to those seeking help with PP&HP in PC [5,8,13,30,31,36,47]. On the other hand, referral and follow-up services provided at the PC centre (e.g., support groups or nutritionists) can act as facilitators [14,27,29,31–33,36,39]. According to patients, health institutions (private and public) do not promote or cover PP&HP because it is not profitable [14,31,33,39–41,46]. Hence, healthcare systems and the pharmaceutical industry promote these strategies. Patients believe that health professionals’ training is focused on treating, not on developing their skills in prevention and promotion of healthy habits [31,40]. In addition, the predominance of the biomedical model means that resources are assigned to diagnosis and treatment. This is associated with patients' opinion that PHC is not suitable for PP&HP [42]. However, those patients who adopted a more holistic model affirmed that PHCs play a crucial role in PP&HP [34].

#### Environment and society

The built environment (i.e., bike lanes, parks or pedestrian paths) contains spaces where healthy activities can be carried out and this motivates patients [29,39,41]. Dietary traditions or other lifestyle factors conditioned by culture have an impact on their behavior [27–29,39,41]. Social norms can promote both healthy and unhealthy lifestyles (e.g., sport and tobacco) [27,28]. Furthermore, social stigma related to some habits (e.g., alcohol consumption or sexual health) can prevent people from acknowledging problems and seeking help to change their habits [27,45]. Mass media is a powerful tool for promoting PP&HP activities although it is sometimes used perversely to promote unhealthy lifestyles [6,38,42,46].

Regarding socioeconomic environment, public policies are perceived as giving little attention to PP&HP. This has an impact on the meso and micro levels but also on factors in the cultural and physical context (e.g., university, mass media or built environment) [6,8,40,42,46]. Finally, working conditions are perceived by some patients as barriers to healthy lifestyles due to the effect on the work/personal-life balance [33].

## Discussion

To the best of our knowledge, this is the first study to identify and synthesize the main barriers and facilitators associated with the implementation of PP&HP as perceived by PC patients. All these factors can be framed in a 4-level ecological model: a) intrapersonal; b) interpersonal; c) institutional; and d) environment and society factors. These 4 levels interact and influence each other and must be taken into account if PP&HP activities are to be successfully implemented and maintained over time in primary care settings.

### Practical Implications

Table 3 summarizes the practical implications of the synthesis results. According to patients, the most numerous factors related to their engagement in PP&PH activities are intrapersonal ones. These factors fit well into the Health Belief Model (HBM) [48]. This model, developed to investigate why people fail to undertake preventive health measures, is one of the most widely employed theories [49]. According to the HBM, assessment of the perceived threat and/or net benefits explains people’s readiness to act and will be influenced by: perceived susceptibility, perceived severity, perceived benefits, perceived barriers, modifying variables, cues to action and self-efficacy. Thus, if patients fail to perceive that one habit is risky or has severe consequences, they have no reason to modify it. Internal and external cues to action (for example, patients' guilt or support by peers) can trigger a lifstyle change. In addition, they must believe that something can be done to prevent the illness and that they have the resources, skills and knowledge to change their behavior. In other words, they should feel that they can control it which calls for information on how to modify unhealthy habits. Accordingly, educating citizens from childhood on health issues using evidence-based information is important. In the recent years, a new strategy called curriculum infusion has shown promising results. Curriculum infusion involves integrating health issues into academic courses at elementary, middle and high school or college levels with the aim of changing attitudes and behaviours [50,51]. For instance, children could study why our body needs different types of food in biology class while they are taught to calculate how many servings of each food group they need to achieve a healthy diet in math. Similarly, in Chemistry, teenagers could study the chemical composition of alcohol and the chemical reactions of alcohol metabolism that explain the various effects on people who drink alcohol. This knowledge can also lead to an increase in the person’s internal locus of control [52,53]. Various studies have shown that people with an internal locus of control are more open to PP&HP activities [54, 55]. Knowledge can also modify erroneus beliefs about the effectiveness and value of PP&HP.

**Table 3:** Practical implications of the synthesis results of the synthesis.

**INTRAPERSONAL**
Curriculum infusion and/or health education
Patient empowerment and training in PP&HP activities
**INTERPERSONAL**
Promote good patient-physician relationships (patient-centered care and shared decision-making)
Training in communication skills and PP&HP activities
Contextualized care (use of community, social and family resources)
**INSTITUTIONAL**
Increased consultation time per patient and PC professional training
Use of tools to contact, motivate and follow-up patients
Promotion of integrated collaborative care (Primary care professionals, specialists and community stakeholders)
**ENVIRONMENT AND SOCIETY**
Mass media campaigns to promote healthy lifestyles
Build environment policies (e.g., access to green areas or public gym’s)
Policies that promote work/personal-life balance and diminish health inequalities
Health education of public and professionals in schools and universities

PC = Primary care; PP&HP = Primary Prevention and Health Promotion

Interpersonal factors play also a key role. The health professional can act as a barrier or enabler, depending on his or her ability to communicate and involve patients in the decision-making process. Similarly, if the relationship is not based on trust and the patients do not rely on their physician, the probability of changing unhealthy behavior is lower. Patients give importance to physicians’ holistic view of their lives including awareness of the patient's particular circumstances. Consequently, health professionals need biopsychosocial training (including communication skills) that must start while they are undergraduates. This is especially important in the new person-centered care model that has been promulgated by the WHO and other health agencies [56]. However, as patients are aware, this would also require changing the structure of an already overwhelmed primary care system. One strategy that would help with time-management would be allowing professionals’ to control their schedule and the provision of visual tools that can help to explain difficult concepts (e.g., disease risk). The use of reminders and follow-up visits, as well as tools for communicating risk and motivating patients, are proven strategies that have been shown to improve adherence [57–59].

Patients also point out that collaborative care is important in the promotion of healthy habits. It is not only the role of the PC physician, other professionals can be involved (e.g., nurses, nutritionists or physiotherapists). They also stress the importance of including other stakeholders from their own family and community (e.g., teachers or gym’ physical trainers) and having access to information about social resources they may need, including free resources. This is closely related to the social prescribing strategy [60]. As such, efforts are needed to provide integrated care and promote a coordinated approach. However, because of fragmented care in most countries, where there is neither vertical (primary, secondary health care), nor horizontal integration (social and health system), this is very difficult. Strategies to promote intra and inter-sector collaboration are needed.

Patients also cite barriers related with the cultural, socio-economic and built environments. The impact of culture on healthy habits is well known [61,62]. Mass media can play a role in promoting good lifestyles but also in maintaining bad ones. Regulatory policies are needed to control their negative influence. These policies have been effective with regard to tobacco [63–65], alcohol and nutrition [66,67]. Public education strategies targeting erroneous beliefs or a particular population can reinforce the impact of these polices [68].

Built environment refers to physical environments that are designed with health and wellness as integral parts of the communities [69]. Patients remark on the importance of having green areas and safer neighborhoods. From their point of view, this can promote physical and mental wellbeing as well as social connectedness and several studies have borne this out [70,71].

Lastly, patients refer to social justice and equity. Lack of opportunities related to socio-economic difficulties (e.g., less education; fewer economic resources limiting access to healthier food or sports centre membership; deprived environment; or work conditions) are considered as major barriers to the adoption of a healthy lifestyle. To avoid this, governments should guarantee the right to health, universal education and adequate working conditions.

These recommendations (detailed in Table 3) are based on the results of the qualitative synthesis and could improve the implementation, acceptability, effectiveness and maintenance of PP&HP activities in primary care. However, little is known on the effectiveness of these strategies and they should be tested.

### Comparison with the factors perceived by primary health care professionals

Results from this review are very similar to the factors perceived by the PHC professionals [9]. Patients and PC professionals acknowledge the influence of the broad context and the basic role of patients’ and professionals’ education. They coincide in pointing out that the final agent who should adopt the behavior is the patient; hence patients’ beliefs, knowledge, attitudes and motivation are vital factors that should be addressed in an effective way. While patients acknowledge a large amount of internal factors, professionals identified more external factors, although the practical implications are very similar.

### Strengths and limitations

A series of limitations should be taken into account. First, we excluded studies focused on children or adolescents as well as those focused on the primary prevention of acute diseases (e.g., vaccination). This could limit the applicability of the measures. However, all chronic conditions, both physical and mental, were considered, including studies focused on the elderly. Limiting the inclusion criteria favors the heterogeneity of the results, their interpretation and the construction of the model. We did not conduct a search of the grey literature and we only included papers written in English, Spanish and Portuguese. The search strategy identified one potential paper in Japanese and another in German that were not included in the synthesis. However, the search strategy prioritized sensitivity over specificity and included electronic searches of four databases and a manual search to minimize the possibility of excluding relevant literature. A notable number of papers were retrieved and, in all likelihood, information reached saturation. One of the papers was written in Portuguese and was only reviewed by one researcher. This could have introduced bias but we prioritized inclusion of the information. With this exception, all searches, selection and synthesis processes were conducted at least in duplicate by independent researchers. Furthermore, researchers from distinct backgrounds (medicine, nursing, pharmacy, psychology and anthropology) participated in the synthesis so enriching the discussion and reinterpretation of the information.

## Conclusions

We carried out a qualitative synthesis of 29 papers centered on PP&HP from the perspective of primary care patients. These results, combined with the article on the barriers and facilitators in PP&PH from the point of view of PC professionals [9] can be used as a framework to develop PP&PH, taking into account the opinions of the main agents involved, promoting personalized care and shared-decision making [72] and thus empowering the patient [73]. When designing new and or complex interventions, qualitative studies involving the key actors in the process should be conducted to identify barriers and facilitators associated with the intervention in real life. The factors identified should be taken into account to elaborate a conceptualized intervention that is acceptable, feasible and sustainable in actual PC practice to ensure the effectiveness of tried and tested PP&HP interventions.

## Supporting Information

S1 Table Detailed search strategies in electronic databases. (DOC)
