# Supplementary material for: Primary Care Patients’ Perspectives of Barriers and Enablers of Primary Prevention and Health Promotion—A Meta-Ethnographic Synthesis
Source: PLoS One. 2015 May 4;10(5):e0125004. doi: 10.1371/journal.pone.0125004 (PMC4418671; doi:10.1371/journal.pone.0125004)
Supplement: S1 Table — (DOC) [file pone.0125004.s001.doc]

**Supplementary Table S1. Detailed search strategies in electronic databases**

Pubmed searches:

Researcher 1:

("Qualitative Research"[Mesh] OR "qualitative study" OR "qualitative research") AND ("Patients"[Mesh] OR "Patients" OR "Patient") AND ("Risk Reduction Behavior"[Mesh] OR "Risk Reduction Behaviour" OR "Lifestyle Risk Reduction" OR "Lifestyle Risk" OR "Risk Behaviour" OR "Risk Reduction" OR "Preventive Health Services"[Mesh] OR "Preventive Health Services" OR "Preventive Medicine" OR "Primary prevention" OR "Early Intervention" OR "Early Medical Intervention" OR "Health Promotion“)

Researcher 2:

(beliefs OR attitude* OR opinion* OR perception* OR barriers OR facilitators) AND ("Patients"[Mesh] OR "Patients" OR "Patient") AND ("Risk Reduction Behavior"[Mesh] OR "Risk Reduction Behaviour" OR "Lifestyle Risk Reduction" OR "Lifestyle Risk" OR "Risk Behaviour" OR "Risk Reduction" OR "Primary Prevention"[Mesh] OR "Health Promotion"[Mesh] OR "primary prevention" OR " health promotion" ) AND ("Qualitative Research"[Mesh] OR "qualitative“)

ISI Web of Knowledge:

Researcher 1:

Topic=(("qualitative study" OR "qualitative research") AND ("Patients" OR "Patient") AND ("Risk Reduction Behavior"[Mesh] OR "Risk Reduction Behaviour" OR "Lifestyle Risk Reduction" OR "Lifestyle Risk" OR "Risk Behaviour" OR "Risk Reduction" OR "Preventive Health Services" OR "Preventive Medicine" OR "Primary prevention" OR "Early Intervention" OR "Early Medical Intervention" OR "Health Promotion")) Timespan=All Years. Search language=English

Researcher 2:

Topic=((beliefs OR attitude* OR opinion* OR perception* OR barriers OR facilitators) AND ("patients" OR "patient") AND ("Risk Reduction Behavior"[Mesh] OR "Risk Reduction Behaviour" OR "Lifestyle Risk Reduction" OR "Lifestyle Risk" OR "Risk Behaviour" OR "Risk Reduction" OR “primary prevention" OR " health promotion") AND ("Qualitative Research" OR "qualitative")) Timespan=All Years. Search language=English.

Global Health:

Researcher 1:

ab ( "qualitative study" O "qualitative research" O “qualitative analysis”) Y af ("Patients” O "Patient") Y af ("Risk Reduction Behavior" O "Risk Reduction Behaviour" O "Lifestyle Risk Reduction" O "Lifestyle Risk" O "Risk Behaviour" O "Risk Reduction" O "Preventive Health Services" O "Preventive Medicine" O "Primary prevention" O "Early Intervention" O "Early Medical Intervention" O "Health Promotion")

Researcher 2:

ab ("Qualitative Research" O "qualitative" O “qualitative analysis”) Y ab ( beliefs O attitude* O opinion* O perception* O barrier* O facilitator* ) Y af ( "Patients” O "Patient") Y af ("Risk Reduction Behavior" O "Risk Reduction Behaviour" O "Lifestyle Risk Reduction" O "Lifestyle Risk" O "Risk Behaviour" O "Risk Reduction" O “primary prevention" O " health promotion")

CINHAL:

Researcher 1:

AB ( "qualitative study" OR "qualitative research") AND TX ( "Patients” OR "Patient") AND TX ("Risk Reduction Behavior" OR "Risk Reduction Behaviour" OR "Lifestyle Risk Reduction" OR "Lifestyle Risk" OR "Risk Behaviour" OR "Risk Reduction" OR "Preventive Health Services" OR "Preventive Medicine" OR "Primary prevention" OR "Early Intervention" OR "Early Medical Intervention" OR "Health Promotion")

Researcher 2:

AB ("Qualitative Research" OR "qualitative") AND AB ( beliefs OR attitude* OR opinion* OR perception* OR barrier* OR facilitator* ) AND TX ( "Patients” OR "Patient") AND TX ("Risk Reduction Behavior" OR "Risk Reduction Behaviour" OR "Lifestyle Risk Reduction" OR "Lifestyle Risk" OR "Risk Behaviour" OR "Risk Reduction" OR “primary prevention" OR " health promotion")
